# Factors associated with caregivers’ contribution to self-care in heart failure

**DOI:** 10.1590/1518-8345.5838.3633

**Published:** 2022-08-12

**Authors:** Ana Maria Miranda Martins Wilson, Glauber Silva Mendes de Almeida, Bruna de Cassia Ferreira dos Santos, Michele Nakahara-Melo, Ana Paula da Conceição, Diná de Almeida Lopes Monteiro da Cruz

**Affiliations:** 1 Universidade de São Paulo, Escola de Enfermagem, São Paulo, SP, Brasil.; 2 Universitatsklinikum Duesseldorf, Kardiochirurgie, Duesseldorf, NRW, Alemanha.; 3 Hospital Municipal Dr Moysés Deutsch, Pronto-Socorro, São Paulo, SP, Brasil.; 4 Universidade Federal de Juiz de Fora, Departamento de Enfermagem Aplicada, Juiz de Fora, MG, Brasil.; 5 Bolsista da Coordenação de Aperfeiçoamento de Pessoal de Nível Superior (CAPES), Brasil.; 6 Instituto Dante Pazzanese de Cardiologia, Unidade de Internação Adulto, São Paulo, SP, Brasil.; 7 Bolsista do Conselho Nacional de Desenvolvimento Científico e Tecnológico (CNPq), Brasil.

**Keywords:** Heart Failure, Caregivers, Self Care, Social Support, Nursing, Cross-Sectional Studies, Insuficiência Cardíaca, Cuidadores, Autocuidado, Apoio Social, Enfermagem, Estudos Transversais, Insuficiencia Cardíaca, Cuidadores, Autocuidado, Apoyo Social, Enfermería, Estudios Transversales

## Abstract

**Objective:**

to analyze the caregiver’s contribution to self-care in heart failure and the predictor variables of this contribution.

**Method:**

a cross-sectional descriptive and analytical study, with the participation of 140 dyads (patients and caregivers). The contribution to self-care was assessed using the Caregiver Contribution to Self-Care of Heart Failure Index. Caregivers and patients were interviewed separately to obtain the data. Multiple linear regressions were used to verify predictor variables of caregiver contribution.

**Results:**

the mean score for contribution to maintenance self-care was 62.7 (SD=7.1), for management, 62.9 (SD=20.4) and for confidence was 63.3 (SD=22.1). The variables number of patient’s medications, caregiver being related to the patient, social perception of caregiver, health-related quality of life of the patient and caregiver’s confidence in contributing to self-care were predictors of caregiver’s contribution to maintenance or management self-care.

**Conclusion:**

the caregiver’s contribution was insufficient. The social support perceived by the caregiver, the type of relationship the caregiver to the patient, the number of medications used by the patient, as well as the caregiver’s confidence in contributing to self-care are variables that should be considered to assess the risk of insufficient contribution of the caregiver.

## Resumo

**Objetivo:**

analisar a contribuição do cuidador para o autocuidado na insuficiência cardíaca e variáveis preditoras desta contribuição.

**Método:**

estudo transversal descritivo e analítico, com a participação de 140 díades (pacientes e cuidadores). A contribuição para o autocuidado foi avaliada por meio do *Caregiver Contribution to Self-Care of Heart Failure Index*. Cuidadores e pacientes foram entrevistados separadamente para obtenção dos dados. A regressão linear múltipla foi utilizada para verificar variáveis preditoras da contribuição do cuidador.

**Resultados:**

a pontuação média de contribuição para o autocuidado de manutenção foi 62,7 (DP=7,1), de manejo, 62,9 (DP=20,4) e na confiança foi 63,3 (DP=22,1). A variáveis número de medicamentos do paciente, cuidador ter parentesco com o paciente, percepção social do cuidador, qualidade de vida relacionada à saúde do paciente e a confiança do cuidador na contribuição para o autocuidado foram preditoras da contribuição do cuidador para o autocuidado de manutenção ou de manejo.

**Conclusão:**

a contribuição do cuidador foi insuficiente. O apoio social percebido pelo cuidador, cuidador ter ou não parentesco com o paciente, número de medicamentos utilizados pelo paciente, bem como a confiança do cuidador em contribuir para o autocuidado são variáveis que devem ser consideradas para avaliar o risco de contribuição insuficiente do cuidador.

## Resumen

**Objetivo:**

analizar la contribución del cuidador para el autocuidado en la insuficiencia cardiaca y las variables predictoras de esa contribución.

**Método:**

estudio transversal descriptivo y analítico, con la participación de 140 díadas (pacientes y cuidadores). La contribución para el autocuidado fue evaluada por medio del *Caregiver Contribution to Self-Care of Heart Failure Index*. Los cuidadores y pacientes fueron entrevistados separadamente para obtención de los datos. La regresión linear múltiple fue utilizada para verificar variables predictoras de la contribución del cuidador.

**Resultados:**

la puntuación media de contribución para el autocuidado de manutención fue 62,7 (DE=7,1), de administración fue 62,9 (DE=20,4) y de confianza fue 63,3 (DE=22,1). Las variables: número de medicamentos del paciente, cuidador tener parentesco con el paciente, percepción social del cuidador, calidad de vida relacionada a la salud del paciente y la confianza del cuidador en la contribución para el autocuidado, fueron predictoras de la contribución del cuidador para el autocuidado de manutención o de administración.

**Conclusión:**

la contribución del cuidador fue insuficiente. El apoyo social percibido por el cuidador, el cuidador tener o no parentesco con el paciente, el número de medicamentos utilizados por el paciente y la confianza del cuidador en contribuir para el autocuidado, son variables que deben ser consideradas para evaluar el riesgo de contribución insuficiente del cuidador.

**Highlights**

(1) Caregivers’ contribution to self-care was insufficient.

(2) Caregivers’ confidence to contribute to self-care was insufficient.

(3) Patient variables influence caregiver contribution to self-care.

(4) Caregivers’ own variables influence their contribution to self-care.

## Introduction

Cardiovascular diseases are the leading cause of death in Brazil; among them heart failure (HF) affects 64.3 million people worldwide and is responsible for the largest number of hospitalizations, which burdens the health system^1^ ^-^ ^3^. Insufficient self-care (SC) is the main cause of hospital admission and readmission of these patients^4^, which is why interventions that promote engagement in SC behaviors become critical to achieve the best possible control of the syndrome.

SC in HF is defined as a naturalistic decision making process that influences the actions that maintain physiological stability, as well as facilitating the perception of symptoms that directs decision making on disease manifestations and treatment effects^5^. This consists of three sequential and interconnected processes: maintenance is the first dimension and refers to health behaviors and treatment adherence; the second is symptom perception, which deals with awareness of physical sensations and analysis of their meaning by listening to the body, monitoring for signs, recognizing, interpreting and classifying symptoms; the third process is management, understood as the response given to symptoms when they occur^5^.

Clinical guidelines advocate that interventions to promote SC should focus on developing the skills a person needs to manage and control symptoms^1^ ^,^ ^6^. Although benefits are described in the literature, the results found among studies of people with HF demonstrate that SC is still far from ideal^5^ ^,^ ^7^ ^-^ ^8^. 

Syndrome symptoms such as fatigue and dyspnea considerably impact the performance of certain SC activities^7^; therefore, family members or informal caregivers can be considered valuable resources for the health care system^9^ ^-^ ^10^. However, interventions that include the caregiver in this approach are still scarce in the literature and in clinical practice^11^. 

The caregiver’s contribution to SC in HF is defined as a person’s provision of time, commitment and support for the benefit of another person with the syndrome who needs to perform his or her SC^11^. The theory of caregiver contribution to SC in HF was developed based on a collection of evidence through studies using the Caregiver Contribution to Self-care of Heart Failure Index (CC-SCHFI) instrument^11^ ^-^ ^12^.

According to the theory, patient and caregiver characteristics directly influence SC; it is assumed that a didactic relationship exists between patient and caregiver, in which patient behaviors elicit contributing behaviors on the part of caregivers, which in turn influence patient behaviors, thus establishing interdependence between patient and caregiver^11^. The quality of the patient-caregiver relationship influences the willingness for mutual contribution within the dyad. 

The caregivers’ contribution is considered a process with actions ranging from recommending a particular behavior or replacing that behavior for the patient. One of the assumptions of the theory is that the caregiver contribution is influenced by factors related to the patient, the caregiver, and the relationship between them^11^. However, there are still few studies that try to verify the caregiver and patient characteristics that influence the caregiver’s contribution and little is known about these relationships in samples of Brazilians.

This study aimed to analyze the caregiver’s contribution to SC in HF and the predictor variables of this contribution.

## Method

### Design, location and time period

This study was a secondary analysis of data obtained in a cross-sectional, descriptive and analytical study conducted with patients with HF and their respective informal caregivers (dyads). The primary study was conducted between September 2019 and February 2021 in outpatient teaching units of specialized cardiology services in the city of São Paulo, SP, Brazil. The two institutions were chosen because they are national references in the care of patients with cardiovascular disorders as well as in teaching and research in the area. Both institutions receive people from all over the state of São Paulo and from other states in Brazil. 

The Strengthening Reporting of Observational Studies in Epidemiology (STROBE) guide from the Enhancing the QUAlity and Transparency Of health Research (EQUATOR) Network was used to organize this report.

### Casuistry

Being under outpatient treatment for HF, have a medical diagnosis of HF, have clinical and cognitive conditions to participate in the study and indicate an informal caregiver (adult person, family member or not, who provides total or partial, non-professional and unpaid care to the person with HF and participates with him in decision making) were the inclusion criteria established for the patients in the study. To be 18 years old or older, to be identified by the patient as his/her main caregiver and to have cognitive conditions to participate in the study were inclusion criteria for the caregivers in the study. Caregivers and patients who met the inclusion criteria were invited to participate in the study and those who agreed to the terms of participation and signed the Free and Informed Consent Term (FICT) were included.

Sampling was by convenience and sample size was calculated at 138 dyads (patient-caregiver) assuming that correlations greater than 0.3 would be detected with type I and II errors of 5% each. The study sample consisted of 140 dyads. Data were collected by face-to-face interview with patients and their primary caregivers who accompanied them to outpatient appointments; patient charts were consulted for clinical data. Patients and caregivers were interviewed separately.

### Instruments

The patient’s SC behavior, the caregiver’s contribution to the patient’s SC, the patient’s confidence for SC and the caregiver’s confidence in his/her contribution to SC were assessed using the Brazilian versions of the Self-Care of Heart Failure Index (SCHFI)^13^ and of the Caregiver Contribution to Self-Care of Heart Failure Index (CC-SCHFI)^13^. The SCHFI is composed of 22 items grouped into three subscales: Maintenance SC (10 items), Management SC (6 items), and Confidence in SC (6 items). The CC-SCHFI is a measure of the caregiver’s contribution to the HC patient’s SC and was developed by mirroring the SCHFI^14^. The instrument also has 22 items in three subscales: contribution to maintenance SC (10 items), contribution to management SC (6 items) and caregiver confidence in contributing to the patient’s SC (6 items). In both instruments, scores for each subscale range from 0-100 points, calculated based on the transformation of pure scores. Higher scores reflect better SC and better contributions to SC and scores greater than or equal to 70 on each subscale are indicative of adequate SC^12^. The Brazilian versions of the SCHFI and the CC-SCHFI obtained good validity and reliability estimates in the adaptation studies for Brazil^13^ ^-^ ^14^. Recent review of SC theory in HF indicates that trust is not a SC behavior, but is a variable that should be included in studies of SC in HF^7^.

Patient’s clinical information, such as: functional class (FC), time of experience with HF, number of medications used, number of comorbidities, HF FC according to the New York Heart Association (NYHA) classification^4^, number of hospitalizations and emergency room visits in the last 12 months and demographic and social information of the patient and caregiver, such as: gender age, education, whether lives with spouse, employment status and whether caregiver and patient live in the same household and type of relationship between patient and caregiver, were obtained by interview or extracted from the patients’ medical records. 

The patient’s health-related quality of life (HRQOL) was assessed by the Brazilian version of the Minnesota Living With Heart Failure Questionnaire (MLHFQ), which has 21 questions about the limitations often associated with the syndrome and how much they prevent patients with HF to live as they would like in the last month. The response options for each question range from zero (no limitation) to five (maximum limitation) in the physical, emotional and other non-dimensional dimensions. The total score is calculated by summing the answers in the 21 items and the scores for each item can range from zero to five points; the overall scale score is from zero to 105 points^15^.

Patients’ knowledge about HF was assessed by a questionnaire adapted and validated for Brazil, which has 14 questions with four alternative answers on topics related to HF care, such as alcohol use, use of drugs, weight control, salt consumption, physical activity, reasons for readmission and general information about HF. The instrument generates an overall score of 100%, which is calculated according to the number of correct answers, in which the higher the number of correct answers, the better is the knowledge about HF^16^. 

The caregiver’s quality of life (QL) was assessed by The World Health Organization Quality of Life (WHOQoL- BREF) in the version adapted for Brazil. The instrument has 26 questions, being two questions of general health perception (number 1 and 2) and the others represent each of the 24 faces related to the perception of quality of life, which encompass the 4 domains that compose the instrument: physical, psychological, social relations, and environment. The answer options for each question follow a Likert scale (from 1 to 5, the higher the score the better the quality of life), with the results ranging from 0 to 100% after calculating and recoding the questions^17^.

The social support perceived by the caregiver was measured by the Medical Outcome Study- Social Support Scale (MOS-SSS), which aims to evaluate to what extent the person counts on the support of others to face different situations in his/her life. The original scale is composed of 19 items that assess five dimensions of social support: material support (four questions), affective (three questions) and emotional (four questions) and information (four questions) and positive social interaction (four questions). For each item, the individual must indicate how often he/she has each type of support within a five-point Likert scale: 1 (“never”); 2 (“rarely”); 3 (“sometimes”); 4 (“almost always”) and 5 (“always”). The overall index is obtained by summing the responses to the 19 items, with higher scores indicating better results regarding social support^18^. 

Caregiver strain was obtained by the Caregiver Role Stress Scale that was developed for Brazil and Colombia. The Brazilian scale has 21 items, with the responses for each item ranging from zero to two. The final score of the scale ranges from zero to 42 and higher scores indicate greater caregiver strain^19^. 

### Data treatment and analysis

The data was analyzed with the aid of the R program (version 4.1.1) by a statistical professional. For quantitative variables, descriptive measures of central tendency and variability were performed; qualitative variables, in turn, were presented in frequencies. 

The normality of the distribution of continuous numerical variables was assessed using the Shapiro-Wilk test. Continuous variables with non-normal distribution were tested for the dispersion of the ranks using Levene’s test. For correlation analyses, Pearson’s correlation coefficient was used and Kendall’s only for ordinal functional class variable. The strengths of the correlations were classified into: <0.30 = weak correlation; ≥0.30 and <0.50 = moderate and >0.50 = strong^20^. 

In the association analyses between the scores of the subscales of the instrument to evaluate the caregiver’s contribution to the SC (CC-SCHFI-Brazilian version) and the socio-demographic variables, the Mann Whitney test or Brunner Munzel test were applied, depending on the normality of the distribution of residuals. To establish the comparison among more than three groups, the variance analysis (ANOVA) or the Kruskal-Wallis test was applied. 

In order to analyze patient and caregiver variables potentially predictive of caregivers’ contribution to SC of patients with HF, multiple linear regression models were built for caregiver contribution to maintenance SC and management SC as outcomes; the variables were defined based on scientific evidence on factors influencing caregiver and patient SC as possible predictors^21^ ^-^ ^22^. Patient confidence for SC and caregiver confidence in their abilities to contribute to SC were included as possible predictors in the model of caregiver contribution to management SC. There was no evidence of multi-collinearity in the predictor variables and generalized variance inflation factor (VIF) of 2 was considered the cut-off point for evidence of multi-collinearity. A significance level of 5% was adopted for all analyses.

### Ethical aspects

The project was approved by the Ethics and Research Committee of the School of Nursing of the University of São Paulo, under CAAE no. 14227119.4.0000.5392 and opinion no. 3.519.739 and by the co-participating institutions: Dante Pazzanese Cardiology Institute (DPIC) under CAAE no. 14227119.4.3003.5462 and opinion no. 3.569.872, and Department of Medicine of Paulista School of Medicine of Federal University of São Paulo (Unifesp) under CAAE no. 14227119.4.3001.5505 and opinion no. 3.497.531 as recommended by the National Health Council Resolution 9 (NHC) No. 466 of 2012, of the Ministry of Health (MH) for evaluation of ethical issues regulating research involving human subjects^23^. Patients and caregivers after being aware of the terms of participation contained in the specific FICT gave their agreement by signing the document.

## Results

A total of 140 patients with HF and 140 informal caregivers, indicated by the patients, participated in the study. Table 1 presents demographic and social characteristics of patients and caregivers.

**Table 1:** Demographic and social characteristics of patients with heart failure (N=140) and their caregivers (N=140). São Paulo, SP, Brazil, 2019-2021

Variables	Patients		Caregivers	
Female gender (n, %)	61	(43.57)	99	(70.50)
Average age (SD*)	64.30	(11.61)	52.03	(13.72)
Average years of schooling (SD*)	4.95	(3.90)	9.30	(4.24)
Living with a spouse (n, %)	97	(69.29)	100	(71.43)
Inactive (retired or pensioner) or not in gainful employment (n, %)	125	(89.28)	64	(45.71)

*SD = Standard deviation.

Of the 140 dyads, 112 (80%) lived in the same household; of the 140 caregivers, 48.57% were spouses, 24.29% were the patients’ daughters, 13.57% were the patients’ father or mother, and the others were sisters (3.57%) or had a relationship other than a kinship with the patient (10.00%). Tables 2 and 3 present descriptive statistics of the other variables of the patients and caregivers, respectively.

**Table 2:** Descriptive statistics of variables of patients with heart failure (N=140). São Paulo, SP, Brazil, 2019-2021

Variables	Statistics		
Mean time to HF* in months (SD^†^, 95%CI^‡^)	113.66	(103.71)	97.56-131.90
Etiology of HF* (n, %)			
Chagasic		41	(29.29)
Hypertrophic		31	(22.14)
Idiopathic		27	(19.29)
Ischemic		22	(15.71)
Other		19	(13.57)
HF functional class* (n, %)			
Class I		33	(23.57)
Class II		74	(52.86)
Class III		31	(22.14)
Class IV		2	(1.43)
Mean left ventricular ejection fraction (SD^†,^ 95%CI^‡^)	0.40	(0.15)	0.37-0.42
Mean comorbidities (SD^†^, 95%CI^‡^)	2.87	(1.47)	2.63-3.12
Mean number of hospitalizations or emergency department visits in the past 12 months (SD^†^, 95%CI^‡^)	1.13	(1.97)	0.86-1.54
Mean number of medications in use (SD^†^, 95% CI^‡^)	6.04	(2.35)	5.65-6.42
**Mean HRQL scores (SD^†^, 95%CI^‡^)**			
Overall (ranges from 0 - 105)	46.78	(21.64)	43.16-50.41
Physical dimension (varies from 0 - 45)	24.03	(11.61)	22.06-25.91
Emotional dimension (varies 0 - 25)	8.99	(6.70)	7.90-10.12
Other questions (ranges 0 - 35)	13.77	(7.97)	12.43-15.11
Mean scores of knowledge about CI* (SD^†^, 95%CI^‡^) (ranges 0 - 100)	43.88	(15.51)	41.27-46.39
Mean scores on self-care in CI* (SD^†^, 95%CI^‡^)			
Maintenance self-care (varies from 0 to 100)	51.69	(13.68)	49.50-53.98
Management self-care (varies from 0 to 100)	60.68	(20.61)	56.71-64.65
Mean scores on confidence for self-care (ranges from 0 to 100) (SD^†^, 95%CI^‡^)	64.52	(18.71)	61.31-67.51

*HF = Heart failure; ^†^SD = Standard deviation; ^‡^95 CI % = 95% confidence interval.

**Table 3:** Descriptive statistics of variables of caregivers of patients with heart failure (N=140). São Paulo, SP, Brazil, 2019-2021

Variables	Statistics		
Average time as caregiver in months (SD*, 95%CI^†^)	78.27	(92.64)	64.90-96.10
Average hours devoted to daily caregiving (SD*, 95%CI95%^†^)	12.02	(8.59)	10.62-13.47
Mean comorbidities (SD*, 95%CI%^†^)	0.88	(1.01)	0.72-1.06
Mean quality of life scores (SD*, 95%CI%^†^)			
Physical domain (range 0-100)	71.48	(14.78)	69.00-73.88
Psychological domain (range 0-100)	70.95	(14.27)	68.38-73.25
Social domain (range 0-100)	68.63	(16.32)	65.92-71.31
Environmental domain (range 0-100)	64.60	(14.34)	62.20-66.99
Mean scores of perceived social support (MOS-SSS^§^) (SD*, 95%CI†)			
Overall (varies from 20 to 100)	74.15	(19.13)	70.83-77.15
Material dimension (varies from 20 to 100)	74.20	(24.70)	69.95-78.10
Affective dimension (varies 20 to 100)	87.06	(20.13)	83.40-90.13
Emotional dimension (varies 20 to 100)	75.95	(23.60)	46.95-48.60
Informational dimension (varies from 20 to 100)	75.70	(22.55)	71.60-79.45
Positive social interaction dimension (varies from 20 to 100)	79.65	(22.55)	75.65-83.15
Mean scores on caregiver role strain (range 0 - 42)	7.99	(7.12)	6.94-9.32
Mean scores on caregiver contribution to self-care in HF^‡^ (SD*, 95%CI^†^)			
Maintenance self-care (varies from 0-100)	62.69	(17.53)	59.65-65.45
Management self-care (ranges 0-100)	62.85	(20.39)	59.34-66.08
Mean scores on confidence in contributing to self-care (ranges 0-100) (SD*, 95%CI^†^)	63.25	(22.15)	59.45-66.80

*SD = Standard deviation; ^†^95%CI = 95% Confidence interval; ^‡^HF = Heart failure; ^§^Medical Outcome Study- Social Support Scale.

Correlation analyses between the scores of caregiver contribution to maintenance SC and caregiver variables showed a positive and weak correlation with the social domain of caregiver QoL with a correlation coefficient (CC) equal to 0.268 and positive and weak correlation with the global score and all dimensions of caregiver perception of social support (the CC ranged from 0.193 to 0.227), except for the informational dimension. There was an association by the One-way ANOVA test between the type of relationship between patient and caregiver with the caregiver’s contribution to the maintenance SC (p=0.026) and the conditions related to being a spouse or daughter had the highest scores compared to the other categories (sister(s), father/mother, other types of relationships). The caregivers who shared the same household with the patient had higher contribution scores to the maintenance SC (64.64 versus 54.88; p=0.046) than those who did not live in the same place verified by means of the Wilcoxon-Mann Whitney test. As for the caregiver’s contribution to the maintenance SC and the patient’s variables, Pearson’s correlation tests showed a positive and weak correlation (CC=0.181) with the number of patient’s comorbidities and with the total HRQL score (CC=0.166) and with the FC of HF (CC=0.136).

Analyses between caregiver contribution to management SC and caregiver variables showed weak or moderate positive correlations with the overall social support score and with all dimensions of social support (CCs ranged from 0.212 to 0.276), with the number of medications used by the patient (CC=0.294), with the total HRQL score (CC=0.272), with the physical dimension of HRQL (CC=0.303) and with the FC of HF (CC=0.215).

Caregiver confidence in contributing to the patient’s SC had positive and weak correlations with the affective (CC of 0.230 and p-value = 0.006) and positive social interaction (CC of 0.168 and p-value = 0.047) dimensions of perceived social support. There was an association between caregiver confidence and the type of relationship between caregiver and patient by means of the Kruskall-Wallis test. Daughter(s) (68.30) and spouse (65.28) scored better (p=0.019) than participants in the other categories. Of the patients’ variables, there was correlation: with the functional class of HF (CC=0.020); with the total HRQL score (CC=0.186) and with the emotional dimension of HRQL (CC=0.212)

Table 4 presents the multiple linear regression model for caregiver contribution to maintenance SC.

**Table 4:** Multiple linear regression model for the caregiver’s contribution to maintenance self-care in heart failure. São Paulo, SP, Brazil, 2019-2021

Variables	Coef^*^	SE^†^	95%CI^‡^	95%CI^‡^	p-value
			Inferior	Superior	
(Intercept)	-2.390	17.490	-37.056	32.275	0.892
**Patient variables**					
Age	-0.074	0.153	-0.378	0.230	0.630
Sex - Male	-0.342	3.319	-6.920	6.236	0.918
Education	0.256	0.393	-0.523	1.035	0.516
Functional class II	-0.615	4.093	-8.727	7.496	0.881
Functional class III	0.065	5.424	-10.685	10.816	0.990
Functional class IV	10.613	12.757	-14.672	35.897	0.407
Syndrome time	0.010	0.016	-0.022	0.041	0.547
Number of medications	1.714	0.653	0.420	3.009	0.010
Comorbidities	1.107	1.034	-0.942	3.157	0.286
Living with caregiver - Yes	1.045	4.295	-7.467	9.557	0.808
Hospitalizations	0.928	0.721	-0.501	2.357	0.201
Knowledge about the syndrome	0.034	0.099	-0.161	0.230	0.730
HRQL^§^ - physical	0.044	0.160	-0.272	0.360	0.782
HRQLRQoS^§^ - emotional	0.255	0.245	-0.231	0.742	0.300
**Caregiver’s variables**					
Age	0.141	0.148	-0.153	0.435	0.344
Sex - Male	-1.552	3.604	-8.696	5.592	0.668
Education	0.297	0.406	-0.509	1.102	0.467
Time as a caregiver	-0.012	0.018	-0.048	0.023	0.491
Hours of daily care	0.022	0.187	-0.349	0.393	0.907
Caregiver is spouse	25.068	7.543	10.119	40.017	0.001
Caregiver is child	25.645	7.654	10.475	40.816	0.001
Caregiver is Sibling	4.055	9.762	-15.294	23.403	0.679
Caregiver is parent	20.127	8.287	3.702	36.552	0.017
Other Relationship	14.402	9.968	-5.353	34.158	0.151
QL^||^ - physical	-0.167	0.120	-0.404	0.071	0.167
Psychological QL^||^	0.127	0.123	-0.117	0.372	0.305
Stress of Caregiver Role	-0.061	0.250	-0.556	0.434	0.807
Social support - global	0.266	0.079	0.109	0.423	0.001

*Coef = Coefficient; ^†^SE = Standard error; ^‡^95%IC = 95% Confidence interval; ^§^HRQL = Health-related quality of life; ^||^QL = Quality of life. R^2^: 0.404; Adjusted R^2^: 0.251.

It is observed that the number of medications (p=0.10) used by the patient with HF was a predictor variable in the caregiver’s contribution to the patient’s maintenance SC, i.e., the higher the number of medications used by the patient, the higher is the caregiver’s contribution to the patient’s maintenance SC. The patient’s bond with the caregiver was a variable that presented itself as predictor for the contribution to the maintenance SC. When the caregiver is a relative, such as spouse (p=0.001), patient’s child (p=0.001), or patient’s parent (p=0.17), the contribution to the maintenance SC increases.

It should also be noted that social support was also a statistically significant predictor variable (p=0.001) and for each unit of increase in the social support scale score, the contribution to the maintenance SC increases by 0.266, on average.

Table 5 presents the multiple linear regression models for caregiver contribution to SC management of patients with HF.

**Table 5:** Multiple linear regression model for the caregiver’s contribution to management self-care in heart failure. São Paulo, SP, Brazil, 2019-2021

Variables	Coef^*^	SE^†^	95%CI^‡^	95%CI	p-value
			Inferior	Superior	
(Intercept)	8.749	20.357	-31.607	49.106	0.668
**Patient variables**					
Age	-0.115	0.177	-0.466	0.235	0.515
Sex - Male	5.383	3.811	-2.172	12.939	0.161
Education	0.295	0.450	-0.598	1.188	0.514
Functional class II	-1.196	4.703	-10.519	8.126	0.800
Functional class III	-1.183	6.289	-13.651	11.284	0.851
Functional class IV	7.520	14.777	-21.774	36.814	0.612
Syndrome time	0.006	0.018	-0.030	0.042	0.742
Number of medications	1.306	0.766	-0.213	2.826	0.091
Comorbidities	-0.052	1.189	-2.409	2.305	0.965
Living with caregiver - Yes	3.877	5.011	-6.056	13.811	0.441
Hospitalizations	-0.326	0.826	-1.963	1.312	0.694
Knowledge about the syndrome	-0.084	0.123	-0.329	0.161	0.497
HRQL^§^ - physical	0.634	0.183	0.271	0.998	< 0.001
HRQLRQoS^§^ - emotional	-0.298	0.288	-0.869	0.273	0.303
Confidence in self care	-0.056	0.093	-0.241	0.130	0.554
**Caregiver’s variables**					
Age	-0.032	0.170	-0.369	0.305	0.851
Sex - Male	0.519	4.126	-7.660	8.698	0.900
Education	0.497	0.465	-0.426	1.420	0.288
Time as a caregiver	-0.017	0.021	-0.058	0.024	0.411
Hours of daily care	-0.173	0.220	-0.609	0.264	0.435
Caregiver is spouse	0.433	9.068	-17.543	18.408	0.962
Caregiver is child	7.471	9.193	-10.752	25.695	0.418
Caregiver is Sibling	11.904	11.402	-10.699	34.506	0.299
Caregiver is parent	1.610	9.803	-17.822	21.043	0.870
Other Relationship	4.674	11.591	-18.305	27.652	0.688
QL^||^ - physical	-0.204	0.137	-0.476	0.069	0.141
Psychological QL^||^	0.106	0.143	-0.179	0.390	0.463
Stress of Caregiver Role	0.467	0.287	-0.102	1.036	0.107
Social support - global	0.293	0.091	0.112	0.474	0.002
Confidence in contributing to self-care	0.326	0.081	0.164	0.487	< 0.001

*Coef = Coefficient; ^†^SE = Standard error; ^‡^95CI% = 95% Confidence interval; ^§^HRQL = Health-related quality of life; ^||^QL = Quality of life. R^2^: 0.439; Adjusted R^2^: 0.282.

However, for the caregiver’s contribution to the SC of management, the patient’s physical HRQL was a predictor variable (p<0.001), showing that in patients with worse physical HRQL evaluations, there is a need for a greater contribution of the caregiver to the SC of management. Also in this construct, social support (p<0.001) proved to be relevant and the higher the social support, the higher the caregiver’s contribution to the SC of patient management. 

Caregiver confidence in contributing to the patient’s SC was considered a predictor variable in this model, revealing that caregivers who are more confident in their process of contributing contribute more to the management of SC.

## Discussion

The informal caregiver is fundamental for the SC of the person with HF^11^ ^,^ ^24^, but the knowledge capable of supporting this statement still needs to advance^10^. The results of this study showed the perception of informal caregivers regarding their contribution to the SC of the person with HF and which caregiver and patient variables independently influence this contribution. Up to the time of finalizing this report, no records of other publications that have described this phenomenon in samples of Brazilians were identified, so this is most likely the first article that does so.

When evaluating the isolated scores of patients and caregivers on SC behaviors and confidence in SC (Tables 2 and 3), it was observed that in all cases the averages were below 70 points and therefore interpreted as insufficient SC. This interpretation needs to be considered carefully because so far there is no empirical basis to support it. This cut-off point is the recommended one for interpreting the SCHFI scores^25^ and, considering that the CC-SCHFI mirrors the SCHFI, we chose to use it in this context, as it has been used in other studies with this same instrument^21^ ^,^ ^25^. 

Although the caregivers’ mean scores were below the cut-off point in SC for maintenance and management and in confidence for SC, they are higher among Brazilian caregivers than among Italian caregivers^21^ ^,^ ^26^ ^-^ ^27^. In an Italian study, the contribution to the maintenance SC had a mean score of 55.9 (most caregivers did not recommend the patient to monitor body weight or to perform physical activities). In the contribution to the management SC, the mean score was 58.4 (most caregivers reported not to quickly recognize the exacerbation of HF symptoms) and, regarding the caregiver’s confidence, the mean score was 56.9, also reflecting low confidence to contribute to the SC of the patient with HF^27^. 

The mean scores of patients with HF in this study are similar to those of other Brazilian or foreign studies that, in general, are also below 70 points indicating insufficient SC^8^ ^,^ ^28^ ^-^ ^31^. The results in Tables 2 and 3 also show that caregivers had higher mean scores than patients in the maintenance SC. In the management SC the same occurs, but the magnitude of the difference is smaller. As for the confidence for the SC, the patients’ mean score was similar to the caregivers’, but these differences were not statistically analyzed. 

In summary, the results discussed so far allow us to state that the studied sample of caregivers, in terms of behaviors contributing to SC, is similar to that of other countries, reiterating the need to recognize, together, the caregiver and the patient with HF as the focus of care offered. SC in HF, as in other chronic conditions, requires hard work that needs to be recognized by the patients themselves^32^ and health professionals in their clinical practices.

Despite recent advances in research on SC in chronic conditions, especially in HF, knowledge production in the area reflects fragmented efforts due to the lack of continuity and abundance of descriptive studies^33^. Therefore, knowing how the problem manifests itself in a given situation is indispensable for the development and evaluation of nursing interventions that encompass the patient and caregiver. 

The regression models in Tables 4 and 5 allowed us to identify some factors that influence the contribution to SC in HF in this sample of caregivers. These results are important to better understand the problem of interest (caregiver contribution to SC in HF) and provide elements to guide the development of interventions that can alleviate or solve the problem. Additionally, including the assessment of factors that influence the caregiver’s contribution to SC in clinical practice may facilitate the early detection of risk of low engagement in SC^34^. Modeling for the *maintenance* SC contribution outcome - that refers to caregiver behaviors that promote patient adherence to treatment and symptom monitoring performed to prevent HF exacerbation^12^ - resulted in the identification of three predictor variables: one from the patient; and two from the caregiver himself (Table 4). 

The addition of medications in use by the patient increases the contribution to maintenance SC. On the caregiver’s part, higher scores in the global perception of social support; caregivers who are spouses, children, sisters or fathers/mothers of the patient contribute more to the maintenance SC when compared to caregivers who are not related to the patient.

Differently from the results of the present study, in another research^34^ the number of medications was a significant predictor variable of the patient’s maintenance SC, but not of the caregiver’s contribution to the management SC. As the syndrome progresses, a greater number of drugs may be necessary for its stabilization and for the management of symptoms, making the pharmacological therapy more complex, which may contribute for the caregiver to be a fundamental support, especially in moments of disease exacerbation, when compliance may be more critical. That is, the fact that the patient uses a greater number of medications can be a sign of greater severity, which can make this interpretation lead the caregiver to contribute more to the maintenance SC. 

It is very likely that among the caregivers who have some kinship with the patient there is a higher proportion of those who live in the same household than among the caregivers who have no such relationship. Living in the same place could favor the incorporation of the caregiver’s contribution behaviors to the maintenance SC to the daily routine, such as reminding the patient to monitor body weight. Interestingly, in another study, the type of relationship was a predictor variable of contribution to management SC and not to maintenance SC^21^. It turns out that in this study^21^ the type of relationship was treated as a dichotomous variable in terms of marital relationship, and the sample of patients and caregivers has some characteristics different from those presented in the present study, particularly, the average age of the patients (in this study it was 64.3 years and in the cited^21^ was 76.26 years) and residence with a spouse (in this 69.29 versus 54.40%), with the caveat that the *married* variable in the comparison study was interpreted here as marital cohabitation. To facilitate the accumulation of evidence on the factors that influence the caregiver’s contribution to SC in HF, it would be productive to have a consensus among researchers in terms of definitions of variables.

Positive perception of social support, which is defined as an exchange of resources between at least two individuals with the goal of improving the recipient’s well-being, has been consistently associated with better caregiver outcomes, including the contribution they make to the SC of people with chronic illnesses, including HF^18^ ^,^ ^21^ ^,^ ^35^ ^-^ ^37^. In this research, the caregiver’s perception of social support positively influenced their contribution to the patient’s maintenance SC and to the management SC. Studies^10^ ^,^ ^19^ point out that caregivers with less social support perceived their caregiving role as less enjoyable and more burdensome than those with more social support. 

In another study^37^, conducted to test a model in which caregiver variables affect their contribution to the SC of management, a positive and direct influence of social support was observed in the contribution to the SC of management, but not to that of maintenance. Comparison of these results is limited because of the differences between the analyses performed. In the present study, the contribution to the maintenance SC as a moderating variable in the relationship between social support and the contribution to the management SC was not tested, which could lead to results similar to those of the aforementioned study^37^. These results confirm that the social support perceived by the caregiver is an important element in his/her contribution to SC and initiatives coming from family members and health professionals that support caregivers in this process can culminate in better SC outcomes.

Caregivers of patients with HF experience different levels of burden and strain^38^. In this study, caregiver role strain was not a predictor of caregiver contribution to SC in HF in any of the models (Tables 4 and 5). However, it is a variable that needs to be considered in studies aiming to test the theory of caregiver contribution to SC in HF, because it has been confirmed as a response variable of social support perceived by the caregiver and of the quality of the personal relationship between patient and caregiver^35^ ^,^ ^39^. 

The patient’s physical HRQL was predictive of the caregiver’s contribution to the SC of *management* in HF as well as the caregiver’s confidence in his or her contribution to the patient’s SC and social support (Table 5), already discussed. This dimension of caregiver contribution addresses the caregiver’s abilities to recognize symptoms, to consider measures to alleviate them and to evaluate the results of such measures^12^. Higher scores on the instrument used for the assessment of patient HRQL in this study indicate worse HRQL^15^. Therefore, the results of the present study indicate that the worse the patient’s HRQL, the better the caregiver’s contribution to SC management. The progression of the syndrome can generate more impact regarding symptoms on the patient’s HRQL and at the same time, require from the caregiver more participation, therefore, more contribution to the SC of patient’s management. 

In line with other research^27^ ^,^ ^40^ and with the theory adopted in this other study^11^, Caregiver confidence was associated with the contribution to the SC of management. Caregiver confidence, according to the theory of caregiver contribution to the SC of the patient with HF^11^, is defined as the caregiver’s belief in his or her ability to help patients with SC in HF. Confidence reflects self-efficacy regarding one’s ability to contribute to SC such as feeling confident about one’s own ability to recognize the symptoms of HF in the patient^11^ and is theorized to mediate the influence between patient, caregiver and patient-caregiver dyad factors and to contribute to the maintenance and management SC^11^. Some studies have sought to test this aspect of the theory^36^, but, there are still gaps related to this knowledge.

This study provides knowledge about the caregivers’ contribution to SC and contributes to improving the theory of the caregiver’s contribution to SC in HF, by adding empirical data on factors potentially associated with the caregivers’ behaviors. The results point to the urgent need to truly bring the caregiver and the person with HF closer to the core of care and nursing interventions. Perhaps, many caregivers of people with HF are not even aware that they are performing this role. Increasingly, health systems have tacitly delegated to people close to the patients, relatives or friends, the responsibility for a significant portion of health care. And this care has increased in quantity and complexity, without, however, informal caregivers having adequate resources and information to meet the demand^10^ ^,^ ^41^. It is necessary, therefore, that there are public policies that can provide resources and necessary support to informal caregivers, including them with patients in the center of health care^42^. 

One of the limitations of this study is its cross-sectional design, which does not allow establishing causal or temporal relationships among the variables. Another limitation is the fact that the sample was composed of users of specialized cardiology services and, therefore, had different characteristics from other populations, limiting the generalization of the results obtained. It is suggested that variables related to the patient-caregiver dyad be considered in other studies on the contribution of the caregiver to the SC of the person with HF, as well as the integration of other outcomes of the person with HF, the caregiver and the patient-caregiver dyad.

## Conclusion

The caregiver’s contribution to self-care of the person with heart failure in the sample studied was insufficient. It was observed that the greater number of medications used by the patient, better social support perceived by the caregiver and the caregiver type of relationship to patient were predictors of better contribution of the caregiver to *maintenance* self-care; whereas, the worse health-related quality of life of the patient, better perception of social support by the caregiver, and greater confidence in their own ability to contribute to self-care of the person with heart failure were predictors of better contribution to *management* self-care.
